# A combined genetic and chemical approach for identifying novel antifungal compounds against *Fusarium graminearum*

**DOI:** 10.1128/spectrum.02961-25

**Published:** 2026-01-22

**Authors:** Soobin Shin, Hyeon Ji Je, Yugyeong Choi, Bomin Kim, Jisu Hong, Juwon Yang, Jae Woo Han, Joon-Ho Lee, Hyun Suk Yeom, Gyung Ja Choi, Hokyoung Son, Hun Kim

**Affiliations:** 1 Department of Agricultural Biotechnology, Seoul National University, Seoul, Republic of Korea; 2 Center for Eco-friendly New Materials, Korea Research Institute of Chemical Technology 65680, Daejeon, Republic of Korea; 3 Department of Medicinal Chemistry and Pharmacology, University of Science and Technology, Daejeon, Republic of Korea; 4 Infectious Diseases Therapeutics Research Center, Korea Research Institute of Chemical Technology 65680, Daejeon, Republic of Korea; 5 Research Institute of Agriculture and Life Sciences, Seoul National University, Seoul, Republic of Korea; 6 Plant Genomics and Breeding Institute, Seoul National University, Seoul, Republic of Korea; Universidade de Sao Paulo, Sao Paulo, Brazil

**Keywords:** antifungal discovery, *Fusarium graminearum*, fungicide target, kinase inhibitor

## Abstract

Chemical fungicides act by targeting specific proteins or pathways, known as modes of action (MoAs). In this study, we established a systematic screening platform using *Fusarium graminearum* gene knockout mutants to evaluate the efficacy of fungicides and identify novel antifungal compounds. Target genes corresponding to major fungicide classes were deleted by homologous recombination, and the resulting mutants exhibited altered sensitivity to the corresponding fungicides compared with the wild-type strain. Deletion of genes encoding known fungicide targets revealed distinct sensitivity profiles, supporting MoA prediction. Using the platform that detects sensitivity differences through OD_₆₀₀_ changes, we examined a chemical library of 2,704 small molecules and identified 8 compounds with activity likely involved in the osmotic signal transduction pathway. Several of these compounds exhibited previously unreported antifungal activity, underscoring their potential as novel scaffolds for future development. Collectively, this platform provides a robust, mechanism-informed system for antifungal discovery, with broad applicability to synthetic agents, natural products, and biocontrol metabolites.

**IMPORTANCE:**

This study provides a perspective on developing strategies to manage fungal pathogens with implications for agriculture and food security. The effectiveness of current fungicides is increasingly limited by resistance, narrow target ranges, and environmental concerns. By combining genetic approaches with chemical screening, this work offers a more systematic way to identify candidate antifungal agents and better understand their possible mechanisms. The framework may also be adapted for use in other areas, including medical and veterinary applications, suggesting potential broader applicability. In addition, the emphasis on exploring new molecular scaffolds supports efforts to develop more sustainable and long-term disease management strategies. Overall, this study contributes useful knowledge and tools for advancing antifungal research and improving approaches to pathogen control.

## OBSERVATION

*Fusarium graminearum* is a major phytopathogenic fungus that causes *Fusarium* head blight (FHB) in cereal crops, including wheat and barley (1). This disease results in severe yield losses and contaminates grains with harmful mycotoxins, posing serious risks to both agriculture and food safety (2). The management of FHB has heavily relied on the application of chemical fungicides targeting essential fungal processes, such as sterol biosynthesis and mitochondrial respiration (3, 4). While these fungicides have contributed to disease control, their intensive and repeated use has led to the emergence of resistant strains, thereby reducing long-term efficacy and raising concerns about sustainable disease management (5). These challenges highlight the urgent need to develop antifungal compounds with a novel mode of action (MoA).

Over the past few decades, the development of fungicides with novel MoAs has progressed more slowly, in part due to overlaps with established mechanisms and the substantial resources required for conventional screening approaches (6). Recent advances in genomics and functional genetics offer new opportunities to establish mechanism-informed discovery platforms. In particular, the concept of chemical genetics, which links small-molecule activity with specific genetic determinants, provides a rational framework for MoA prediction (7). Leveraging gene deletion mutants as biological reporters can be effective in linking chemical activity with molecular targets (8). These advances highlight the potential of integrative, genetics-based screening strategies to accelerate antifungal discovery.

**Fig 1:**
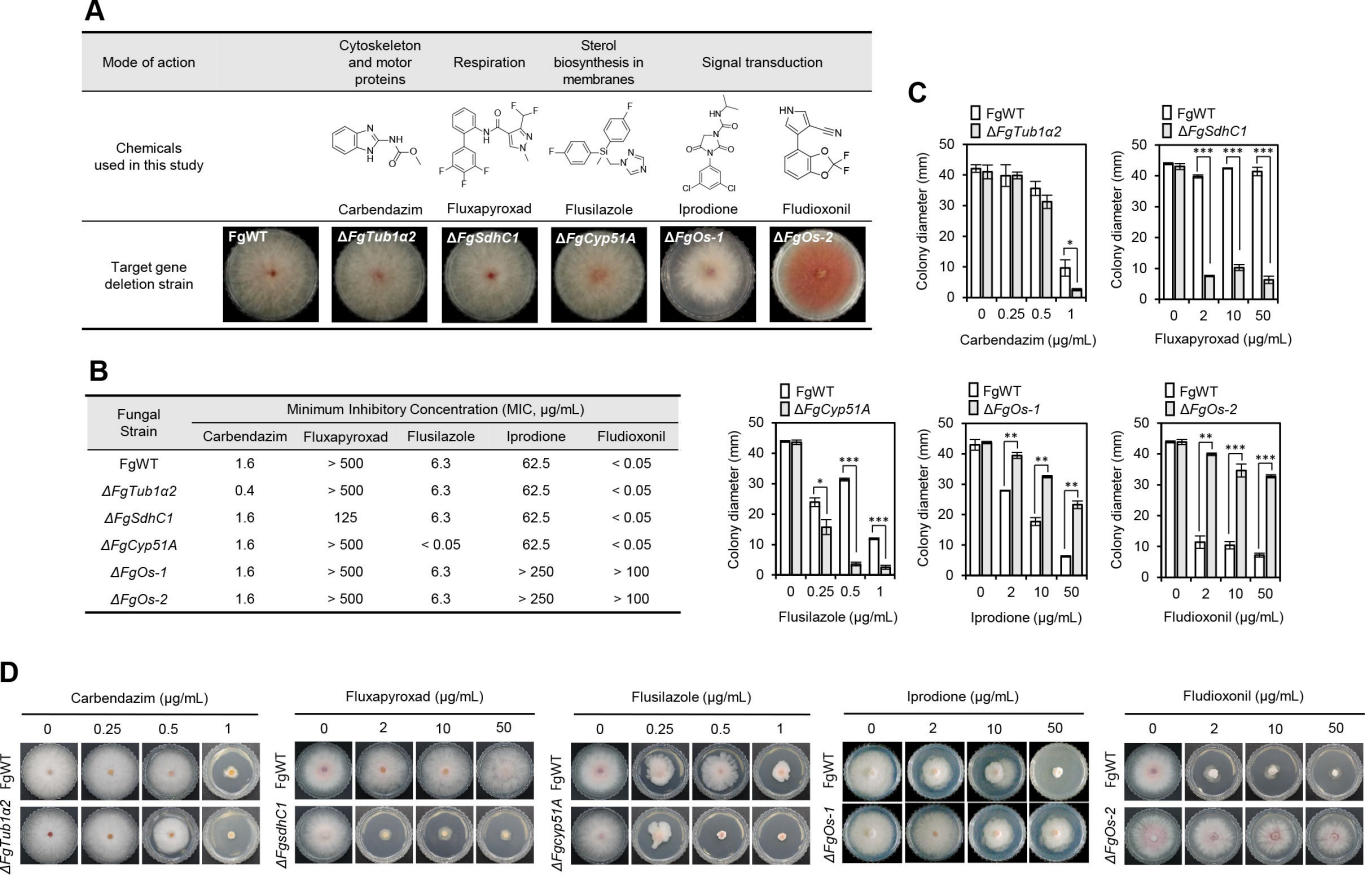
Antifungal activity of fungicides against *F. graminearum* strains. (**A**) Overview of the five fungicides used in this study. The chemical structures, known MoAs, and the corresponding *F. graminearum* mutants associated with each chemical are summarized. The *F. graminearum* wild type (FgWT) and targeted deletion strains were grown on CM medium, and photographs were taken after 5 days of incubation at 25°C. (**B**) Minimum inhibitory concentrations (MICs) were determined using the microtiter broth dilution method. Conidial suspensions (1 × 10^4^ conidia/mL of CM medium) of each strain were treated with fungicides using twofold serial dilutions starting from 100 µg/mL (carbendazim, flusilazole, and fludioxonil), 250 µg/mL (iprodione), or 500 µg/mL (fluxapyroxad). MICs were defined as the lowest concentration at which complete growth inhibition was observed after 24 h of incubation. (**C**) Effect of chemical fungicides on the mycelial growth of *F. graminearum* strains. Colony diameters were measured 3 or 4 days after inoculation on CM medium supplemented with each fungicide. Data are shown as the mean ± SD. Asterisks indicate a statistically significant difference in mean values (*P < 0.05; **P < 0.01; and ***P < 0.001 based on Student’s *t*-test). (**D**) Representative culture plates for the differential growth of *F. graminearum* strains grown on CM medium supplemented with each fungicide. Photos were taken 3 or 4 days post-inoculation.

Gene deletion mutants represent a valuable resource for evaluating differential sensitivity to fungicides. By comparing the responses of wild-type and mutant strains, it is possible to infer the biological pathways affected by antifungal compounds (9, 10). In this study, we generated targeted gene deletion mutants of *F. graminearum*, including the primary target genes of major fungicide classes, and assessed their sensitivity to representative fungicides. To delete fungicide target genes in *F. graminearum*, we selected the genes known to serve as the principal targets of each fungicide class, including α-tubulin (*FgTub1α2*), succinate dehydrogenase complex subunit C (*FgSdhC1*), sterol 14α-demethylase (*FgCyp51A*), and osmosensor histidine kinases (*FgOs-1* and *FgOs-2*) (Fig. 1A; Fig. S1; Table S1) (Fungicide Resistance Action Committee; https://www.frac.info). We note that other orthologs (or paralogs) within these target pathways have secondary or redundant functions, which allows deletion of the primary target genes without loss of essential cellular processes (11). Using homologous recombination with a hygromycin resistance cassette, we successfully deleted *FgTub1α2, FgOs-1*, and *FgOs-2* and confirmed correct integration by diagnostic PCR (Fig. S1). For *FgSdhC1* and *FgCyp51A*, previously generated strains Δ*FgSdhC1* and Δ*FgCyp51A* were used (12, 13). Consequently, five targeted gene deletion mutants were obtained for subsequent antifungal assays.

To evaluate the impact of the target gene deletion on fungicide sensitivity, we determined the minimum inhibitory concentrations (MICs) of fungicides against *F. graminearum* wild type and gene deletion mutants using conidial suspensions. The MIC assays revealed that Δ*FgTub1α2*, Δ*FgSdhC1*, and Δ*FgCyp51A* mutants exhibited increased sensitivity to their corresponding fungicides—carbendazim, fluxapyroxad, and flusilazole, respectively—compared with the wild-type strain (Fig. 1B). In contrast, the Δ*FgOs-1* and Δ*FgOs-2* mutants exhibited enhanced resistance to both iprodione and fludioxonil (Fig. 1B). Given that FgOs-1 and FgOs-2 are components of the mitogen-activated protein (MAP)/histidine kinase signaling pathway, deletion of either gene likely alters the response to both fungicides, even though iprodione and fludioxonil have been reported to primarily target FgOs-1 and FgOs-2, respectively (14). Moreover, no cross-resistance was observed among the mutants against the five tested fungicides (Fig. 1B). When the effects of fungicides on mycelial growth were further assessed, our results showed that Δ*FgTub1α2*, Δ*FgSdhC1*, and Δ*FgCyp51A* mutants exhibited increased sensitivity to their respective fungicides compared with the wild type. In contrast, Δ*FgOs-1* and Δ*FgOs-2* mutants exhibited increased resistance, consistent with the conidial suspension assays (Fig. 1C and D). These results suggest that differences in fungicide sensitivity between the wild-type and gene deletion strains can be exploited to elucidate the MoA of novel antifungal agents and identify potential lead compounds for fungicide development.

Most currently used fungicides, such as sterol biosynthesis inhibitors (e.g., azoles) and respiration inhibitors (e.g., strobilurins and succinate dehydrogenase inhibitors), target highly conserved enzymatic processes that are essential for fungal growth and survival (15). Although these MoAs have been effective, their extensive and long-term use has led to widespread resistance in many fungal pathogens (16). In contrast, fungicides targeting the MAP/histidine kinase signaling pathway (e.g., iprodione and fludioxonil) act through a fundamentally different mechanism, disrupting environmental stress signaling and osmoregulation rather than directly inhibiting primary metabolic enzymes (17). Despite the potential of this pathway as a fungicide target, only a limited number of compounds have been developed.

**Fig 2:**
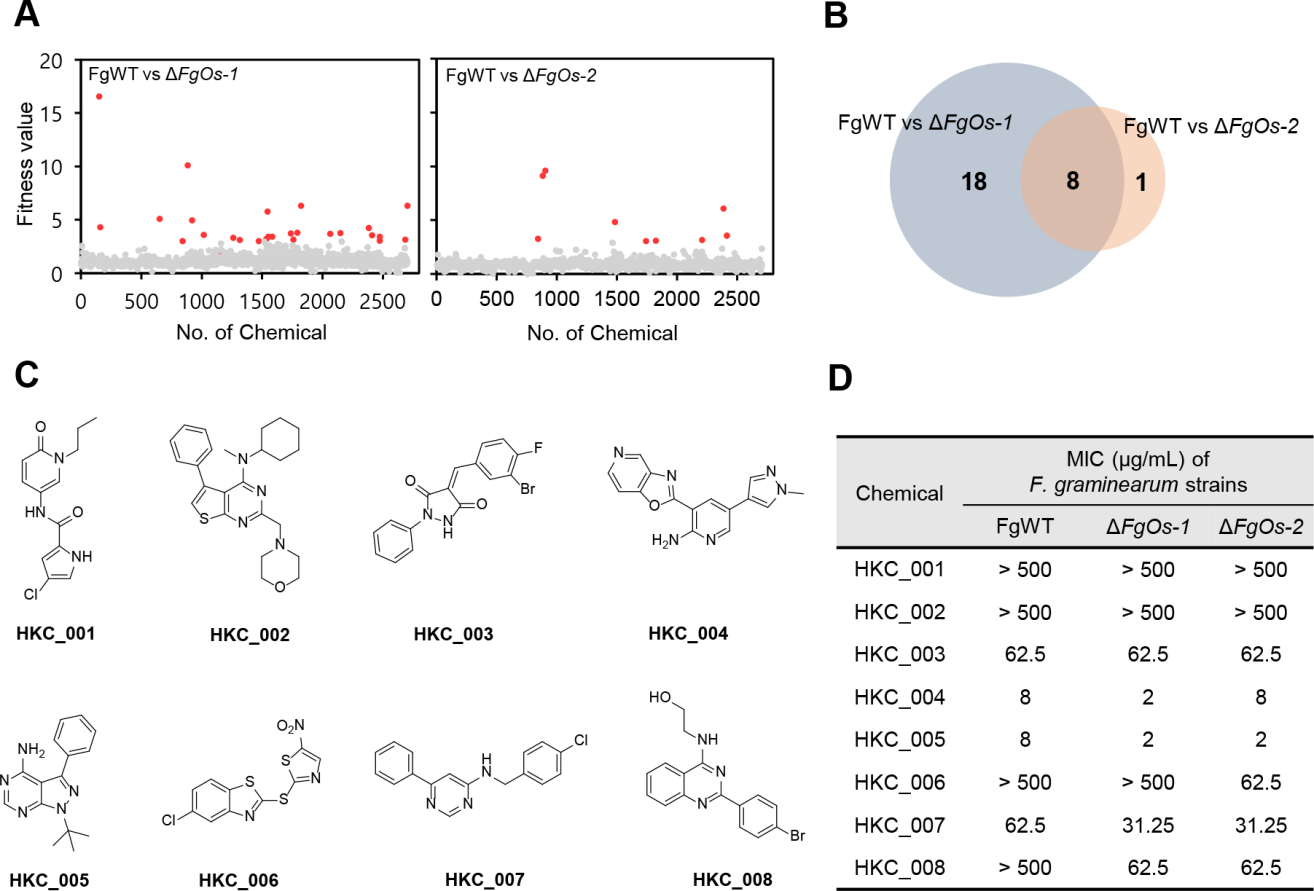
Sensitivity-based screening for the identification of MAP/histidine kinase inhibitors against *F. graminearum*. (**A**) Comparative antifungal activity of 2,704 compounds screened against *F. graminearum* wild-type strain (FgWT) and targeted-deletion strains (Δ*FgOs-1* and Δ*FgOs-2*). Compounds represented by red dots exhibit a fitness value greater than 3. (**B**) Number of compounds showing differential sensitivity between FgWT and Δ*FgOs-1,* and between FgWT and Δ*FgOs-2*, respectively. (**C**) Chemical structures of eight candidate compounds identified as potential inhibitors for the MAP/histidine kinase in *F. graminearum*. (**D**) MICs of eight candidate compounds against *F. graminearum* strains. MICs were determined using the microtiter broth dilution method. Conidial suspensions (1 × 10^4^ conidia/mL of CM medium) of each strain were treated with fungicides using twofold serial dilutions starting from 500 µg/mL. MICs were defined as the lowest concentration at which complete growth inhibition was observed after 24 h of incubation.

Herein, we screened 2,704 small molecules using the Δ*FgOs-1* and Δ*FgOs-2* mutants based on the differential sensitivity between the wild-type and gene deletion strains (Fig. 2A; Table S2). From the initial screening, 26 and 9 compounds exhibited altered antifungal activity in the Δ*FgOs-1* and Δ*FgOs-2* mutants, respectively, with 8 compounds commonly affecting both mutants (Fig. 2B and C). The convergence of these eight compounds on both mutants strongly suggests that they may interfere with a shared signaling pathway, most likely the osmotic histidine kinase cascade, and thus represent promising candidates for further investigation. However, subsequent MIC assays against the wild-type, Δ*FgOs-1*, and Δ*FgOs-2* strains revealed that three compounds (HKC_001, 002, and 003) exhibited identical MIC values against all strains, contradicting the initial screening results (Fig. 2D). Therefore, we shifted our focus to the remaining compounds whose MIC values were altered in the mutants, as these more reliably reflected differential sensitivity linked to the gene deletion. Nevertheless, compounds HKC_004 to HKC_008 exhibited reduced MIC values in Δ*FgOs-1*, Δ*FgOs-2*, or both mutants (Fig. 2D). Given that the Δ*FgOs-1* and Δ*FgOs-2* mutants are less sensitive to iprodione and fludioxonil, we aimed to identify compounds that conferred increased MIC values in the mutants compared with the wild type. However, no such compounds were identified during the initial screening.

Among the five compounds exhibiting differential antifungal sensitivity, three (HKC_004, HKC_007, and HKC_008) have, to our knowledge, not been previously associated with biological activity, suggesting that they may represent novel structural classes with unexplored antifungal potential. In contrast, HKC_005 (4-amino-1-tert-butyl-3-phenylpyrazolo[3,4-d]pyrimidine) and HKC_006 (5-chloro-2-((5-nitrothiazol-2-yl)thio)benzo[*d*]thiazole) have been reported as a kinase inhibitor and an ATP-competitive inhibitor, respectively (18, 19). In particular, HKC_005 has been shown to inhibit the oncogenic tyrosine kinase Src, a central regulator of cell invasion and metastasis, as well as phospholipase D, a lipid-metabolizing enzyme critical for oncogenic signaling (20, 21). More recently, HKC_005 was identified as a selective inhibitor of MKK3, which regulates tumor cell proliferation and survival (22). Beyond mammalian systems, HKC_005 has also been reported to target calcium-dependent protein kinases in *Plasmodium falciparum* and *Toxoplasma gondii*, indicating broad-spectrum activity against kinases involved in stress responses and survival (23, 24). These findings raise the possibility that HKC_005 has broad-spectrum kinase inhibitory potential and may interfere with the MAP/histidine kinase-mediated signaling in *F. graminearum*. Given the promising antifungal activity of HKC_004 and HKC_005 against *F. graminearum* wild type, these compounds may serve as lead structures for fungicide development through derivative synthesis and optimization.

Beyond the findings of this study, the identified candidate compounds can be further examined using computational approaches such as molecular docking or virtual screening to predict their potential binding targets within the osmotic signal transduction pathway. In addition, the genetics-based sensitivity data generated here provide a mechanism-rich resource that could support advanced computational methods for improving compound prioritization. Therefore, integrating these experimental results with *in silico* analyses may accelerate the discovery of pathway-specific modulators and broaden the applicability of our platform.

## SUPPLEMENTAL MATERIAL

Table S1, Table S3, Fig S1, and detailed Materials and Methods spectrum.02961-25-s0001.pdf Table S1: strain information. Table S3: primer list. Fig. S1: strategies for target gene deletion and PCR confirmation. Additional Materials and Methods.

Table S2 spectrum.02961-25-s0002.xlsx Initial screening results.

ASM does not own the copyrights to Supplemental Material that may be linked to, or accessed through, an article. The authors have granted ASM a non-exclusive, world-wide license to publish the Supplemental Material files. Please contact the corresponding author directly for reuse.
