# Supplementary material for: A combined genetic and chemical approach for identifying novel antifungal compounds against Fusarium graminearum
Source: Microbiol Spectr. 2026 Jan 22;14(3):e02961-25. doi: 10.1128/spectrum.02961-25 (PMC12955473; doi:10.1128/spectrum.02961-25)
Supplement: Table S1, Table S3, Fig S1, and detailed Materials and Methods — Table S1: strain information. Table S3: primer list. Fig. S1: strategies for target gene deletion and PCR confirmation. Additional Materials and Methods. [file spectrum.02961-25-s0001.pdf]

Supplemental Material

A combined genetic and chemical approach for identifying novel antifungal compounds against *Fusarium graminearum*

Soobin Shin[1†], Hyeon Ji Je[2†], Yugyeong Choi[2], Bomin Kim[2,3], Jisu Hong[2], Juwon Yang[1], Jae Woo Han[2,3], Joon-Ho Lee[4], Hyun Suk Yeom[2], Gyung Ja Choi[2,3], Hokyoung Son[1,5,6]*, Hun Kim[2,3]*

[1]Department of Agricultural Biotechnology, Seoul National University, Seoul, Republic of Korea

[2]Center for Eco-friendly New Materials, Korea Research Institute of Chemical Technology, Daejeon, Republic of Korea

[3]Department of Medicinal Chemistry and Pharmacology, University of Science and Technology, Daejeon, Republic of Korea

[4]Infectious Diseases Therapeutics Research Center, Korea Research Institute of Chemical Technology, Daejeon, Republic of Korea

[5]Research Institute of Agriculture and Life Sciences, Seoul National University, Seoul, Republic of Korea

[6]Plant Genomics and Breeding Institute, Seoul National University, Seoul, Republic of Korea

[†]Soobin Shin and Hyeon Ji Je contributed equally to this work.

*Correspondence: Hokyoung Son (hogongi7@snu.ac.kr) and Hun Kim (hunkim@krict.re.kr)

Table S1. *Fusarium graminearum* strains used in this study

Strain	Genotype	Deleted gene locus	Source or reference
Z-3639	Wild type	-	1
ΔFgTub1α2	*ΔFgTub1α2::Hyg*	FGSG_00397	In this study
ΔFgSdhC1	*ΔFgSdhC1::Hyg*	FGSG_01981	2
ΔFgCyp51A	*ΔFgCyp51A::Gen*	FGSG_04092	3
ΔFgOs-1	*ΔFgOs-1::Hyg*	FGSG_16781	In this study
ΔFgOs-2	*ΔFgOs-2::Hyg*	FGSG_09612	In this study

REFERENCES

1. Bowden RL, Leslie JF. 1999. Sexual recombination in *Gibberella zeae*. Phytopathology 89:182–188.
2. Kim B, Nguyen MV, Park J, Kim YS, Han JW, Lee J-Y, Jeon J, Son H, Choi GJ, Kim H. 2024. Edeine B_1 produced by *Brevibacillus brevis* reduces the virulence of a plant pathogenic fungus by inhibiting mitochondrial respiration. mBio 15:e0135124.
3. Son H, Seo YS, Min K, Park AR, Lee J, Jin JM, Lin Y, Cao P, Hong SY, Kim EK, Lee SH, Cho A, Lee S, Kim MG, Kim Y, Kim JE, Kim JC, Choi GJ, Yun SH, Lim JY, Kim M, Lee YH, Choi YD, Lee YW. 2011. A phenome-based functional analysis of transcription factors in the cereal head blight fungus, *Fusarium graminearum*. PLoS Pathog 7:e1002310.

Table S3. Primers used in this study

Primer	Sequence (5´to 3´)	
FgTub2-5F	GAAGAGATCAGGGCGTTACAG	
FgTub2-5R	tccactagctccagccaGCTAAGACGAGAGTTTGAAGTTTG	
FgTub2-3F	cagaagaatagcttagcagagtctGCATGATGCGACTTTGTGTAAT	For FGSG_00397
FgTub2-3R	TGTGTTTGTTGGCAACTTGG	deletion
FgTub2-5N	CCAAACAGACAACCGACATAAC	
FgTub2-3N	GCTGAGGGAGATACTACAGAGA	
FgOs1-5F	TGGTGTTTGTTTGCACCAC	
FgOs1-5R	tccactagctccagccaCAGCTCCAATTCAAGCTTACG	
FgOs1-3F	cagaagaatagcttagcagagtctTGAGAAGTCTGACCAACTAACC	For FGSG_16781
FgOs1-3R	GCATCTTGATCGCTTCACTTC	deletion
FgOs1-5N	GCTGGTATACATTACGTGGTAGAG	
FgOs1-3N	CTACTCTTCTCAAAGCACCAGAG	
FgOs2-5F	TACGCTTTGATGGGACTGAC	
FgOs2-5R	tccactagctccagccaGCGAGAAGTTTGAGGTTGAAAG	
FgOs2-3F	cagaagaatagcttagcagagtctCTTTACCCAGGTCTCCAACTAAC	For FGSG_09612
FgOs2-3R	ACATCGTGCATCATACTTCCTC	deletion
FgOs2-5N	GACCAACCAGACACAAGAGTAT	
FgOs2-3N	GAGGAAAGGGCAAAGGAGAA	
HYG_F	TGGCTGGAGCTAGTGGA	Hyg resistance
HYG_R	AGACTCTGCTAAGCTATTCTTCTG	marker
HYG_SF	GGAGACGCTGTCGAACTTT	Deletion
HYG_SR	AAAGTTCGACAGCGTCTCC	confirmation

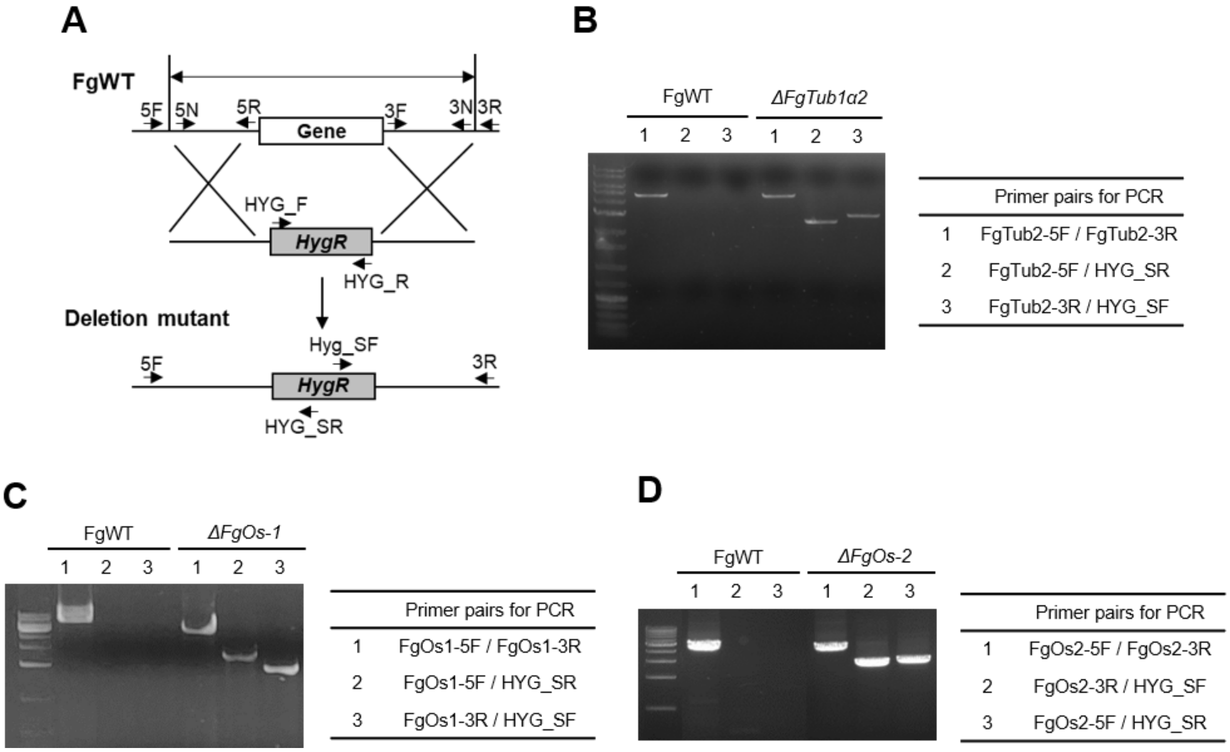

Figure S1. Strategies for target gene deletion in *Fusarium graminearum* and PCR confirmation. (A) Schematic representation for gene deletion by which the gene in *F. graminearum* wild-type strain was replaced with hygromycin resistance gene (*HygR*) by homologous recombination. (B, C, and D) PCR confirmation for the deletion of target genes (B, *FgTub1α2*; C, *FgOs-1*; and D, *FgOs-2*) in *F. graminearum*. Three primer pairs were used, and the results showed distinct PCR amplifications between FgWT and deletion strains.

MATERIALS AND METHODS

Fungal strains and culture conditions

The *Fusarium graminearum* wild-type strain Z-3639 was used as the parental strain for targeted gene deletion. All strains were stored as mycelial suspensions in a 20% glycerol solution at -80°C. Carboxymethyl cellulose (CMC) medium was used for conidium induction, and complete medium was used to measure mycelial growth, as previously described in the Fusarium laboratory manual (4). For conidial suspensions, five fresh mycelial plugs of each *F. graminearum* strain were inoculated in 50 mL of CMC medium and incubated at 25°C for 5 days on a rotary shaker (150 rpm) (5). Mycelial growth was assessed by measuring the colony diameter of the strains grown on complete medium at 25°C, 3–4 days after inoculation.

Genetic manipulations and fungal transformations

Gene deletion mutants were generated using the double-joint PCR method (6). Briefly, the 5′ and 3′ flanking regions of each target gene were amplified from the genomic DNA of strain Z-3639, and the hygromycin resistance gene cassette (*HYG*) was amplified from pGEMT_Hyg (7). Three fragments were fused by double-joint PCR, and the final constructs were amplified using the nested primers. The resulting amplicons were transformed into protoplasts of the Z-3639 strain as previously described (8). Transformants were selected on hygromycin-containing medium and confirmed by PCR. Primer sequences used in this study are listed in Table S2.

In vitro antifungal activity assay

In vitro antifungal activity was evaluated by determining minimum inhibitory concentrations (MIC) using the microtiter broth dilution method (9). Briefly, conidial suspensions (5×10^4 conidia/mL) of each strain were treated with fungicides dissolved in dimethyl sulfoxide (DMSO) by 2-fold serial dilutions. The final DMSO concentration did not exceed 1% (v/v), and a 1% DMSO treatment was used as a negative control. MICs were defined as the lowest concentration that completely inhibited growth after 24 h incubation. To investigate mycelial growth inhibition, a 5 mm mycelial disc of each strain was inoculated onto a complete medium supplemented with fungicides. Colony diameters were measured at 3–4 days post-inoculation. All experiments were conducted twice, with three replicates for each treatment.

Chemical library screening and resource

A chemical library of 2,704 compounds was obtained from the Chemical Bank of Korea Research Institute of Chemical Technology (Daejeon, Republic of Korea). Each compound was supplied as 5 μL of an approximately 5 mM solution in DMSO. For the initial screening, 1 μL of each compound (5 mM) was added to 99 μL of conidial suspension (5×10^4 conidia/mL) in 96-well plates. A 1% DMSO treatment was used as a negative control. After an 18-h incubation, the optical density at 600 nm (OD_{600}) of each well was measured using a microplate spectrophotometer (Bio-Rad, Hercules, CA, USA). To evaluate the differential sensitivity, the

fitness value (FV) was calculated for each strain as follows: FV = (final OD_{600} of the DMSO treatment – initial OD_{600} of the DMSO treatment) / (final OD_{600} of the drug treatment – initial OD_{600} of the drug treatment). The relative fitness values of deletion mutants were expressed as $FV_{\Delta FgOs\text{-}1}/FV_{FgWT}$ and $FV_{\Delta FgOs\text{-}2}/FV_{FgWT}$. The selected compounds HKC_001, 002, 003, 004, 006, and 007 were purchased from Acros Pharmatech Limited (Montreal, Canada); HKC_005 from BLD Pharmatech (Shanghai, China); and HKC_008 from Enamine US Inc. (Monmouth Junction, NJ, USA).

REFERENCES

4. Leslie JF, Summerell BA. 2008. The *Fusarium* laboratory manual. John Wiley & Sons.
5. Cappellini R, Peterson J. 1965. Macroconidium formation in submerged cultures by a nonsporulating strain of *Gibberella zeae*. Mycologia 57:962–966.
6. Yu JH, Hamari Z, Han KH, Seo JA, Reyes-Domínguez Y, Scazzocchio C. 2004. Double-joint PCR: A PCR-based molecular tool for gene manipulations in filamentous fungi. Fungal Genet Biol 41:973–981.
7. Kim B, Nguyen MV, Park J, Kim YS, Han JW, Lee J-Y, Jeon J, Son H, Choi GJ, Kim H. 2024. Edeine B_1 produced by *Brevibacillus brevis* reduces the virulence of a plant pathogenic fungus by inhibiting mitochondrial respiration. mBio 15:e0135124.
8. Son H, Seo YS, Min K, Park AR, Lee J, Jin JM, Lin Y, Cao P, Hong SY, Kim EK, Lee SH, Cho A, Lee S, Kim MG, Kim Y, Kim JE, Kim JC, Choi GJ, Yun SH, Lim JY, Kim M, Lee YH, Choi YD, Lee YW. 2011. A phenome-based functional analysis of transcription factors in the cereal head blight fungus, *Fusarium graminearum*. PLoS Pathog 7:e1002310.
9. Wiegand I, Hilpert K, Hancock RE. 2008. Agar and broth dilution methods to determine the minimal inhibitory concentration (MIC) of antimicrobial substances. Nat Protoc 3:163–175.
